# Physiological differences between histologically defined subdivisions in the mouse auditory thalamus

**DOI:** 10.1016/j.heares.2010.12.016

**Published:** 2011-04

**Authors:** Lucy A. Anderson, Jennifer F. Linden

**Affiliations:** a Ear Institute, University College London, London WC1X 8EE, UK; b Department of Neuroscience, Physiology & Pharmacology, University College London, London WC1E 6BT, UK

**Keywords:** APT, anterior pretectal nucleus, CF, characteristic frequency, CYO, cytochrome oxidase, LGN, lateral geniculate nucleus, MGB, medial geniculate body, Pol, lateral part of the posterior thalamic nucleus, PSTH, post-stimulus time histogram

## Abstract

The auditory thalamic area includes the medial geniculate body (MGB) and the lateral part of the posterior thalamic nucleus (Pol). The MGB can be subdivided into a ventral subdivision, forming part of the lemniscal (primary) auditory pathway, and medial and dorsal subdivisions, traditionally considered (alongside the Pol) part of the non-lemniscal (secondary) pathway. However, physiological studies of the auditory thalamus have suggested that the Pol may be more appropriately characterised as part of the lemniscal pathway, while the medial MGB may be part of a third (polysensory) pathway, with characteristics of lemniscal and non-lemniscal areas. We document physiological properties of neurons in histologically identified areas of the MGB and Pol in the anaesthetised mouse, and present evidence in favour of a distinctive role for medial MGB in central auditory processing. In particular, medial MGB contains a greater proportion of neurons with short first-spike latencies and high response probabilities than either the ventral or dorsal MGB, despite having low spontaneous rates. Therefore, medial MGB neurons appear to fire more reliably in response to auditory input than neurons in even the lemniscal, ventral subdivision. Additionally, responses in the Pol are more similar to those in the ventral MGB than the dorsal MGB.

## Highlights

► We compared subdivisions of medial geniculate body and posterior thalamic nucleus. ► Latencies were shortest in medial subdivision, longest in dorsal subdivision. ► Response probability was highest in medial and ventral subdivisions. ► Some ventral subdivison and posterior thalamic neurons showed late “rebound” firing. ► Results support tripartite organisation of ascending auditory pathways.

## 1 Introduction

**Fig. 1:**
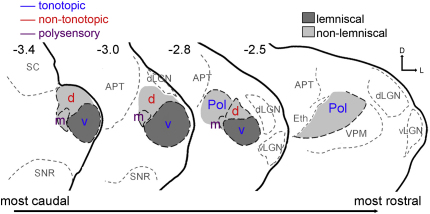
Line drawings of four coronal sections through a typical mouse thalamus to show the relative position of the MGB subdivisions and the Pol. Borders have been ascertained on the basis of CYO staining; outlines are left unfinished where a precise border could not be determined in this animal. Auditory areas are outlined in black dashed lines; non-auditory areas are shown by grey dashed lines to help orientate the reader. Areas thought to belong to the lemniscal pathway are shown in dark grey and areas thought to belong to the non-lemniscal pathway are shown in light grey (grey fills are representative only, and are not intended to show definitive boundaries). Blue lettering indicates tonotopic areas, non-tonotopic areas are represented by red lettering and polysensory areas are shown in purple. Sections have a thickness of 40 μm, numbers at the top of each section give an indication of the section’s distance (in mm) behind Bregma. Abbreviations: v, ventral MGB; m, medial MGB; d, dorsal MGB; Pol, lateral part of the posterior thalamic nucleus; APT, anterior pretectal nucleus; d/vLGN, dorsal/ventral lateral geniculate nucleus; Eth, ethmoid thalamic nucleus; PF, parafascicular thalamic nucleus; SC, superior colliculus; SNR, reticular substantia nigra; VPM, ventral posteromedial thalamic nucleus. Orientation bar, D = dorsal, L = lateral.

The mammalian auditory thalamus is comprised of three main areas: the medial geniculate body (MGB), the lateral part of the posterior thalamic nucleus (Pol) and the auditory sector of the reticular nucleus (Jones, 1985). The MGB is considered the principal nucleus of the auditory thalamus and can be further subdivided into at least three major subdivisions (the ventral, dorsal and medial MGB) on the basis of anatomy, histochemistry and physiological response properties in a number of species (Anderson et al., 2007 [guinea pig]; Anderson et al., 2009b [mouse]; Calford, 1983 [cat]; Cruikshank et al., 2001 [mouse]; Gonzalez-Lima and Cada, 1994 [mouse]; Hackett et al., 1998 [monkey]; Morest, 1964 [cat]; Winer et al., 1999 [rat]). The relative positions of these subdivisions in the mouse MGB are shown in Fig. 1.

The auditory thalamus is an obligatory relay for information passing to the auditory cortex. As with the other sensory modalities, the ascending auditory thalamocortical pathways can be anatomically subdivided into two largely separate, parallel channels. The ventral MGB is considered to be part of the primary (lemniscal) pathway (dark grey in Fig. 1); that is, it receives strong projections from the central nucleus of the inferior colliculus and projects to layers III and IV of the primary auditory cortex (Oliver and Huerta, 1992 [review]; Winer, 1992 [review]; Winer et al., 2005 [review]). Traditionally, other parts of the auditory thalamus, the dorsal and medial MGB and the Pol, which receive input from all parts of the inferior colliculus and other brain areas, were thought to be part of the secondary (non-lemniscal) system (light grey fill Fig. 1) projecting more strongly to the non-primary (secondary) auditory cortices and terminating across layers I, II, III/IV and VI (Jones, 1985; Kimura et al., 2003; Winer et al., 2005).

The lemniscal and non-lemniscal pathways have classically been considered to engage in different auditory functions, with the lemniscal pathway providing a high-fidelity, primary-like representation of sound features (de-Ribaupierre, 1997), while the non-lemniscal pathway supplies more context-dependent information, containing for example neurons which show ability to detect change (Anderson et al., 2009b; Kraus et al., 1994), which are sensitive to multimodal stimuli and reward stimuli (Komura et al., 2005; Komura et al., 2001) or which undergo rapid retuning following behavioural conditioning (Edeline, 1999; Hu, 2003 [review]). Basic physiological response properties recorded from the ventral and dorsal MGB are consistent with this view. In the ventral MGB, neurons are tonotopically organised and exhibit greater fidelity in response timing and frequency tuning than the majority of cells in the non-lemniscal dorsal MGB (Anderson et al., 2007; Calford, 1983; Calford et al., 1983; Redies and Brandner, 1991; Rodrigues-Dageff et al., 1989). However, while the high-fidelity lemniscal versus context-dependent non-lemniscal generalisation seems to hold true for the majority of neurons in the ventral and dorsal MGB, it is an oversimplification for the other subdivisions. Recordings from the medial MGB (typically also considered a non-lemniscal auditory thalamic area) not only show the full range of responses seen in both the dorsal and ventral MGB (Aitkin, 1973; Anderson et al., 2007; Calford, 1983; Calford et al., 1983; Rouiller et al., 1989) but include some neurons which exceed the capabilities of those in the ventral MGB, with extremely short response latencies (Anderson et al., 2006; Rodrigues-Dageff et al., 1989) and temporal following to very high rates (Anderson et al., 2005; Rouiller et al., 1981; Wallace et al., 2007). Likewise, the Pol has traditionally been considered part of the non-lemniscal system (Jones, 1985), but has been described as very similar to the ventral MGB in terms of its physiological properties, containing tonotopically organised neurons with short-latency, narrowly-tuned responses (Imig and Morel, 1985).

To overcome the pitfalls of imposing the anatomical lemniscal/non-lemniscal classification onto areas with overlapping physiological response patterns, a series of three parallel pathways has been proposed in the cat based on physiological as well as anatomical data (Andersen et al., 1980a; Andersen et al., 1980b; Calford and Aitkin, 1983; Rodrigues-Dageff et al., 1989; Rouiller and de-Ribaupierre, 1985; Rouiller et al., 1989). These three distinct parallel pathways − the tonotopic, non-tonotopic and polysensory projections (de-Ribaupierre, 1997; Rouiller, 1997) − originate in the midbrain and each encompasses one of the three major MGB subdivisions. The tonotopic pathway is comprised of the ventral MGB and Pol (blue lettering in Fig. 1), while the non-tonotopic pathway includes the dorsal MGB (red lettering in Fig. 1). The third, the polysensory pathway, encompasses the medial MGB (purple lettering in Fig. 1), which receives auditory input not only from all parts of the inferior colliculus but also from the ventral lateral lemniscus (Whitley and Henkel, 1984) and the cochlear nucleus (Anderson et al., 2006; Malmierca et al., 2002; Strominger et al., 1977), as well as sensory information from many non-auditory structures, including the amygdala, dorsal column nuclei, vestibular nuclei, trigeminal nuclei and spinal cord (Bordi and LeDoux, 1994b; Linke, 1999; Rouiller et al., 1989; Winer, 1992 [review]).

Here, we compare physiological responses of neurons in each of the major MGB subdivisions and the Pol of the mouse. Our data suggest that the tripartite subdivision of the ascending thalamocortical pathways previously proposed in the cat represents a more appropriate description of the mouse auditory thalamus than the more simplistic dichotomy between high-fidelity lemniscal and context-dependent non-lemniscal pathways. Moreover, our results indicate that, despite having low spontaneous rates, neurons in the medial MGB can fire more reliably in response to auditory input than other auditory thalamic neurons, even those in the lemniscal ventral MGB.

## 2 Methods

### 2.1 Animals

Twenty-six adult male CBA/Ca mice, 6-24 weeks of age, were used as subjects in this experimental study. Mice were anaesthetised with ketamine and medetomidine, prepared for recording and monitored using procedures similar to those described in Linden et al. (2003). A craniotomy approximately 2 mm in diameter, centred 2 mm lateral to midline and 3 mm caudal to bregma, was performed on either the left-hand (12) or right-hand (14) side, enabling vertical access to the MGB. The cortical surface was kept moist by regular application of warmed saline. All experiments were performed in accordance with the United Kingdom Animal (Scientific Procedures) Act of 1986.

### 2.2 Recording strategy

Extracellular recordings were made across all MGB subdivisions using custom-made tungsten-in-glass electrodes (tip sizes 10–20 μm, impedance typically 1–2 MΩ (Bullock et al., 1988)). Electrodes were positioned stereotaxically, and advanced using a hydraulic probe drive (FHC 50-12-1C) which was controlled from outside the sound-attenuated booth (Neurocraft MCM/MCU). The motor controller was zeroed as the tip of the microelectrode touched the cortical surface (confirmed microscopically and by an acoustic change in the electrode signal) to ensure consistency across penetrations. In all penetrations the electrode was moved down 2200 μm below the cortical surface, then left to stabilise for ∼10 min. The first penetration was typically made rostro-medially within the craniotomy. Subsequent penetrations were made laterally every 200 μm until no further auditory activity was recorded at that particular rostro-caudal position; the electrode was then returned to a position 100 μm medial to the most medial position which yielded auditory activity and moved 200 μm caudally. As before, subsequent penetrations were made every 200 μm in a lateral direction. If a potential recording site overlapped with a large blood vessel that particular site was abandoned and the electrode was moved to the next position. This strategy for making penetrations ensured even spacing of electrode tracks, which also facilitated histological reconstruction of recording sites. On average, each successful penetration yielded 4 good recording sites, and 3 successful penetrations were made per animal.

### 2.3 Auditory stimuli

Free-field auditory stimuli were directed at the animal’s ear contralateral to the craniotomy, and a sound-attenuating plug was placed in the ipsilateral ear. Prior to the start of each experiment, acoustic stimuli were calibrated with a microphone positioned as close as possible to the opening of the animal’s auditory canal. The sound system frequency response was flat to within ± 2 dB from 2 to 90 kHz. Neurons responsive to auditory stimuli were located using either a 50 μs click or broadband noise stimuli 100 ms in duration, presented at variable intensities. Once an auditory response had been established, the neuron’s response to 100 repetitions of a 50 μs click presented at ∼ 60 dB SPL with an inter-stimulus interval of 500 ms was recorded. To estimate each neuron’s frequency-intensity response area, characteristic frequency (CF), minimum threshold and bandwidth, three repetitions of tone pips at variable frequencies and intensities were presented in a sequential manner (2–75 kHz, 5–80 dB SPL with tones presented at least every 1/5 octave in 5 dB steps; tones had a 5 ms rise/fall time, 100 ms duration and were presented with an inter-stimulus interval of 800 ms).

### 2.4 Data analysis

#### 2.4.1 First-spike latency

The click stimulus was used to provide a measure of neuronal response time as latencies recorded using this broadband measure are less susceptible than tone response latencies to variation with stimulus rise time, and are therefore more useful for cross-study comparisons. Neurons were considered to be responsive to the click if a post-stimulus time histogram (PSTH, compiled from 100 repetitions of the click using bin-widths of 0.5 ms) showed a peak in firing following the click presentation which exceeded two times the standard deviation of the neuron’s spontaneous rate. We defined the term *first-spike latency* for each responsive neuron as the median latency of spike times over 100 click presentations, calculated from the PSTH using bin-widths of 0.5 ms.

#### 2.4.2 Spontaneous rate

The spontaneous firing rate of each neuron was calculated from both the first 4 ms of recording to the click stimulus (i.e. after presentation of the click and before a response was observed) and the mean response across all frequencies presented at 20 dB below the neuron’s minimum threshold. There was no significant difference between these two measures (Kruskal-Wallis test, *p* > 0.1).

#### 2.4.3 Temporal response pattern

To establish whether a neuron’s firing rate returned to pre-response levels following the presentation of the click, we carried out a randomisation test comparing the cell’s spontaneous rate with its firing rate ∼250 ms after click onset. For each cell, the firing rate was calculated from a 4 ms window falling within a “late” period, 150–400 ms after the click, in which firing appeared to be elevated; this value was then compared to the spontaneous firing rate measured in the 4 ms prior to the click response. The average trial-by-trial difference in firing rates between these two periods was then compared to the differences obtained when the assignment of firing rates to late vs. spontaneous intervals on each trial was randomised 10,000 times, to test the null hypothesis of no difference between late firing and spontaneous rates. Neurons were considered to be showing increased late firing if the firing rate in the late window was significantly different from that in the spontaneous interval at *p* < 0.01 in this test. All significant population results using late intervals chosen on a cell-by-cell basis were also significant when a fixed late interval was used.

#### 2.4.4 Frequency-intensity response area

In order to directly compare our data from the mouse with published data from other species we categorised the frequency-intensity response areas into response shapes described in previous MGB studies: primary-like (V-shaped), narrow, broad, and multipeaked (Anderson et al., 2007; Morel et al., 1987; Rodrigues-Dageff et al., 1989; Rouiller et al., 1989). In addition, we also identified level-dependent and inhibitory response shapes which were probably categorised as “atypical” in previous studies. We did not assign a characteristic frequency to neurons with inhibitory response shapes. All response area categorisation was based upon bandwidth measurements and qualitative assessments by an observer blind to the histological location of the recording. Bandwidth measurements were taken 10, 20 and 40 dB above the neuron’s minimum threshold. If the neuron did not show a consistent response to pure-tone stimuli it was classed as untuned.

#### 2.4.5 Statistics

We used non-parametric statistics throughout to avoid the need to make assumptions regarding the distribution of our data. While some of our data distributions did appear to be Gaussian, non-parametric tests provided sufficient power that it was not necessary to use stronger constraints in our statistical tests. On the only occasion where we report a p value between 0.01 and 0.05, the data were not normally distributed; thus requiring the use of the non-parametric test.

### 2.5 Localisation of recording sites

Discrete electrolytic lesions were created by passing current through the electrode (5 μA for 5 s). For each rostro-caudal position, electrolytic lesions were created at the beginning and end of both the most medial track which yielded data, and a second lateral track a known distance from the first (no less than 300 μm). The presence of two or more lesions created a fixed distance apart within an electrode track (as controlled by the hydraulic microdrive) and between electrode tracks (controlled by the stereotaxic manipulator) allowed for estimation of tissue shrinkage and cutting angle. Histological reconstruction of all recording sites could then be accomplished using recorded stereotaxic co-ordinates and the estimated corrections for variations due to histological processing.

**Fig. 2:**
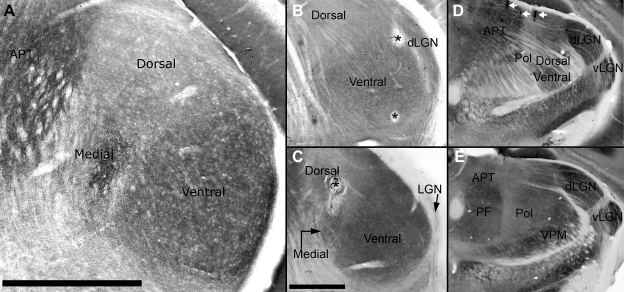
Histological identification of the auditory thalamic subdivisions using cytochrome oxidase. A, coronal section through the MGB stained for cytochrome oxidase. The paler stained dorsal MGB is bounded ventrally by the deeper staining ventral and medial MGB and medially by the anterior pretectal nucleus (APT). B, coronal section through the ventral and dorsal MGB showing two electrolytic lesions (indicated by asterisks) made in the same track where abrupt physiological changes occurred. This physiological judgement was confirmed by the location of the lesions near the borders of the area of higher CYO expression corresponding to the ventral MGB. This section is more rostral than A, and the lateral geniculate nucleus can be observed forming the lateral boundary of the ventral and dorsal MGB. The dLGN is clearly separated from the ventral and dorsal MGB by a band of pale stained fibres. C, asterisk indicates the centre of a large electrolytic lesion made in the centre of dorsal MGB. This section is rostral to A and caudal to B; the very caudal edge of LGN can be observed in the white matter. D, section through the rostral MGB; ventral and dorsal MGB remain just visible and the caudal portion of the paler staining Pol starts to appear within the fibre tracts. White arrowheads at the top of the section indicate stained red blood cells resulting from damage caused by electrode tracks. E, section ∼ 400 μm rostral from the same animal as D, to indicate the volume changes of the Pol on moving rostrally. Scale bar in A = 1 mm, scale bar in C applies to B and C = 500 μm, D and E = 1 mm. Abbreviations as Fig. 1.

The lesions were created once physiological recording in that track had ceased, and prior to moving to a new track. Physiological responses were recorded at each potential lesion site prior to making the lesion; the presence of the lesion was confirmed by the cessation of activity after the current had been passed. There was no physiological or histological evidence that lesions disrupted recording in subsequent tracks, and the lesions had a diameter no greater than 75 μm (smaller than the distance between penetrations). Since the distance between recording sites (within or between electrode tracks) could reliably be controlled, the electrolytic lesions were also used to locate potential sites of interest in a track, for example positions where the physiological response properties of recorded neurons abruptly changed (Fig. 2B).

Electrolytic lesions were visualised in individual brain sections stained for cytochrome oxidase (CYO) using a Zeiss AxioPlan 2 Imaging microscope (magnification x25–x200). Histological procedures were as described in Anderson et al. (2009a). Once the absolute co-ordinates of each recording site had been corrected for tissue shrinkage and cutting angle, the position of each neuron was assigned to the appropriate subdivision as defined according to CYO distribution. On the basis of CYO distribution, the original Golgi scheme of dividing the mouse MGB into three main subdivisions, the ventral, dorsal and medial MGB, can be reliably applied (Gonzalez-Lima and Cada, 1994). If the position of a recording site was ambiguous the data were not included in this study.

## 3 Results

### 3.1 Cytochrome oxidase distribution in the CBA/Ca mouse auditory thalamus

In coronal sections stained for CYO (Fig. 2), a large, moderately stained oval region occupied the ventral and lateral region of the MGB. This region corresponds to the ventral MGB. Gradients of CYO expression could be observed within the ventral MGB both across the medio-lateral axis where staining intensity was greatest ventrolaterally, and along the rostro-caudal axis where the staining intensity decreased towards the rostral and caudal poles. Towards the rostral pole the ventral MGB is bounded laterally by the lateral geniculate nucleus (LGN, see Fig. 2B, D and E) and medially by the Pol (Fig. 2D and E).

The medial MGB showed strong CYO expression which was comparable to the strongest expression observed in the ventral MGB. There was no obvious gradient of CYO expression in the medial MGB along any axis. The medial MGB was separated from the ventral MGB by a band of paler staining and was not obviously present at the rostral pole of the MGB.

The dorsal MGB showed very low CYO expression which clearly differentiated it from the ventral and medial MGB. In some animals a patch of higher CYO expression was observed along the medial edge of the dorsal MGB (dorsal to, but clearly separated from the medial MGB). This darker staining area is likely to correspond to the suprageniculate nucleus; however, unlike the major ventral, dorsal and medial MGB subdivisions, borders for the suprageniculate nucleus were not reliably apparent in all animals.

The caudal extent of the Pol is located medial to the dorsal and medial MGB and lateral to the anterior pretectal nucleus. The caudal border of the Pol was sometimes difficult to determine due to the number of fibre tracts which run through this area. More rostrally, the volume of the Pol increases to fill the space left by the MGB and more discrete boundaries can be observed (compare Fig. 2D and E). At the very caudal end of the Pol, CYO expression was difficult to ascertain due to the number of non-staining fibre tracts, however more rostrally the Pol showed intermediate CYO expression, comparable to the weaker staining seen at the rostral and caudal poles of the ventral MGB. In sections stained for CYO, the Pol in the mouse can be observed to extend rostro-caudally for ∼1.3 mm, however typically we only recorded from the caudal 500 μm.

### 3.2 General characteristics of the recordings

Extracellular physiological responses were recorded from 326 units (181 well-isolated single units and 145 multiunit clusters). Subdivision assignments were determined post-hoc based on histological reconstruction of electrode tracks from electrolytic lesions: 162 neurons were assigned to the ventral MGB, 50 to the dorsal MGB, 82 to the medial MGB and 32 to the caudal portion of the Pol. Physiological responses were recorded from all parts of the dorsal MGB. However, since the suprageniculate nucleus could only be clearly identified in some animals, we compared the responses of neurons from the 6 dorsal MGB tracks which had clearly passed through the suprageniculate to the responses of 19 other neurons which had been recorded more laterally in the dorsal MGB. There were no significant differences between these two populations in any of the physiological properties recorded (Kruskal-Wallis test, *p* > 0.1), thus the recordings were pooled here for analysis of dorsal MGB response properties. There was no significant difference in response properties between right and left hemispheres either across the whole auditory thalamus or its subdivisions (Kruskal-Wallis test, *p* > 0.1). Moreover, similar physiological trends were observed among single units and multiunits separately, so results are reported here for pooled single-unit and multiunit data. All results are summarised in Table 1.

### 3.3 Response to 50 μs click

#### 3.3.1 Latency of response

The response to a 50 μs click was recorded from 316 units; of these 96% (304/316) gave responses to a 50 μs click from which an unequivocal latency could be measured. (This proportion may have been augmented by the use of a broadband search stimulus.) The individual subdivisions all contained a high proportion of cells which responded well to the click: ventral MGB 97.5%, medial MGB 97.6%, dorsal MGB 92% and Pol 93.7%.

**Fig. 3:**
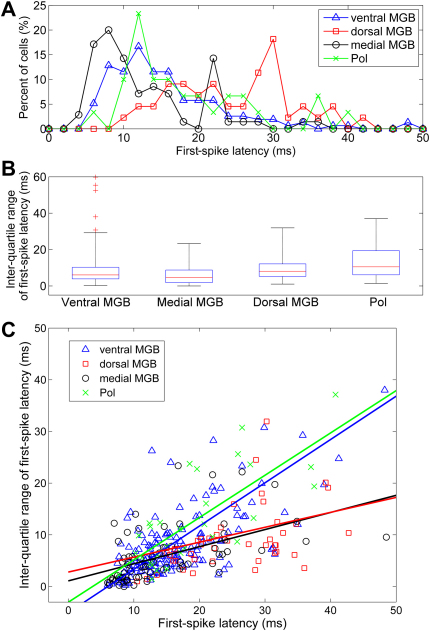
First-spike latencies. A, line plot showing the percentage distribution of first-spike latencies across the ventral, dorsal & medial MGB, and Pol. B, box and whisker plots of inter-quartile range for first-spike latency, for all four subdivisions. On each box, the central mark is the median, the edges of the box are the 25th and 75th percentiles and the maximum whisker length indicates three times the inter-quartile range; data points outside this range are marked as outliers (+). C, scatterplot comparing inter-quartile range of first-spike latency to first-spike latency. Solid lines indicate two-dimensional least-squares linear fits to the data from each subdivision.

The distribution of first-spike latencies in response to a 50 μs click varied both within and across the auditory thalamic subdivisions as shown in Fig. 3A and Table 1. First-spike latencies were significantly shorter in the medial MGB than in all other subdivisions (Kruskal-Wallis test, *p* < 0.01 in all cases), and were significantly longer in the dorsal MGB than in all other subdivisions (Kruskal-Wallis test, *p* < 0.01 in all cases). Indeed, the distribution of first-spike latencies for the dorsal MGB peaked over 18 ms later than the distributions for the other subdivisions. Response latencies were similar, however, in the ventral MGB and Pol (Kruskal-Wallis test, *p* > 0.1).

Fig. 3B shows the inter-quartile range of first-spike latencies recorded across the auditory thalamic subdivisions. The distribution of inter-quartile ranges was only weakly different between all subdivisions in multiple-group comparison (Kruskal-Wallis test, *p*< 0.05, > 0.01), although pairwise comparison between the two most different subdivisions revealed that the range of response latencies was significantly smaller in medial than dorsal MGB (Kruskal-Wallis test, *p* < 0.01). There was no significant correlation between first-spike latency to click and firing rate to the click stimulus or tonal response properties (minimum response threshold, CF, bandwidth (Spearman’s rank correlation test, *p* > 0.1 for all cases)). This was also true for each subdivision if the population was divided into two groups corresponding to either long (> 20 ms) or short (< 20 ms) response latencies.

Variability in first-spike latency (as quantified by inter-quartile range of first-spike latency) increased with increasing first-spike latency in all subdivisions. However, the relationship between inter-quartile range of first-spike latency and first-spike latency differed between subdivisions, as shown in Fig. 3C. In this scatterplot, colour-coded lines indicate two-dimensional least-squares linear fits to the data from each subdivision. (Note that two-dimensional least-squares linear fitting is more appropriate here than linear regression, because a measured rather than independent variable is plotted along the x-axis.) Slopes of lines fit to data from ventral MGB and Pol were similar, as were slopes of lines fit to data from dorsal and medial MGB, but there was a significant difference between the slopes for those two groups (slope ± SE: ventral 0.84 ± 0.06, Pol 0.82 ± 0.13; dorsal 0.29 ± 0.12, medial 0.33 ± 0.07). This result demonstrates that the relationship between first-spike latency and first-spike latency variability was different between ventral MGB and Pol on the one hand, and dorsal and medial MGB on the other.

#### 3.3.2 Probability of response

**Fig. 4:**
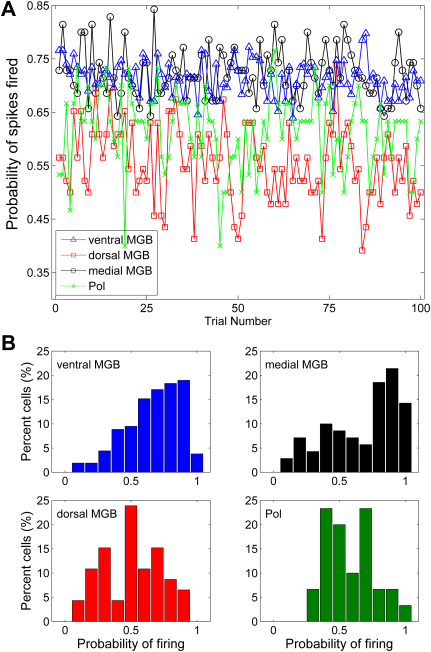
Response probabilities. A, mean probability of response (averaged across the population) for each subdivision plotted against each repetition of the click. B, distribution of the probability of spiking in response to the click averaged across all trials for each subdivision.

To investigate the reliability of the response to a click across the 100 repetitions of the stimulus, the mean probability that one or more spikes would be elicited (averaged across the population recorded from each subdivision) was plotted for each repetition of the click stimulus (see Fig. 4A). Fig. 4A shows on average there was ∼72% probability that at least one spike would be elicited by the stimulus for neurons in the medial and ventral MGB, with no significant change with increasing trial number. Conversely, on average, for cells in the Pol there was only a 62% probability of eliciting at least one spike to each click, while in the dorsal MGB this probability was reduced to 55% although again there was no change with trial number. There was no indication that the presence or absence of a spike being fired on a preceding trial influenced the likelihood of firing in the next trial.

Fig. 4B shows the distribution of probability of spiking averaged across all trials within each subdivision. A high proportion of neurons in both medial and ventral MGB had high (80% or greater) probability of firing to the click stimulus (no significant difference between medial and ventral MGB, Kruskal-Wallis test, *p* > 0.1). In the case of the medial MGB, ∼14% of neurons showed 100% probability of firing (compared to 4% of ventral MGB neurons); in contrast in the dorsal MGB and Pol, probability of firing was centred around 50–60% (Kruskal-Wallis test, no significant difference between dorsal MGB and Pol (*p* > 0.1) but significantly lower probability than both medial and ventral MGB (*p* < 0.01)). This decreased probability of firing in the dorsal MGB or Pol is unlikely to be due to adaptation or suboptimal presentation rates as Fig. 4A shows there was no obvious decrement in response to later trials. Indeed, the probability of firing to the last five trials was not significantly different from the probability of firing to the first five trials for any of the subdivisions (Kruskal-Wallis test, *p* > 0.1). Across all subdivisions, those neurons which showed the highest probability of firing to the click stimulus were also those which had the highest firing rates; however there was no obvious correlation between the neuron’s probability of firing and its tonal response properties (CF, bandwidth, or minimum threshold).

#### 3.3.3 Spontaneous rates

**Fig. 5:**
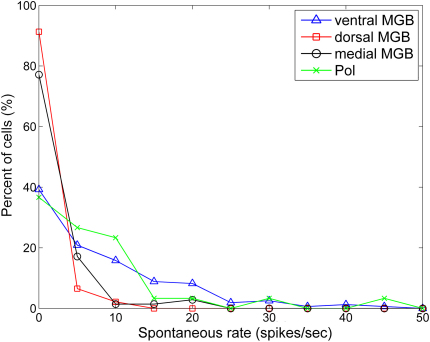
Spontaneous firing rate distributions for all subdivisions.

Fig. 5 shows the distribution of spontaneous rates across the different subdivisions. The medial and dorsal MGB subdivisions were found to have the greatest proportion of cells which showed virtually no spontaneous firing (77% and 91% respectively), and the maximum spontaneous firing rate in these subdivisions was 15 Hz and 11.5 Hz respectively. In comparison, the distribution of spontaneous rates in the ventral MGB and Pol was broader, with fewer cells showing very low spontaneous firing and maximum spontaneous rates exceeding 30 Hz in both cases.

**Fig. 6:**
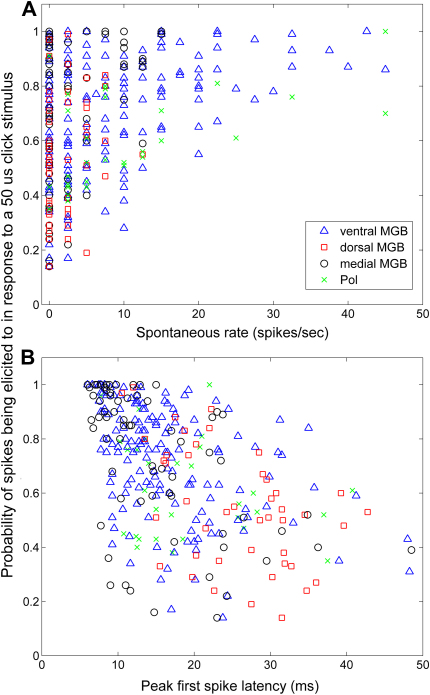
Scatter plots showing the probability of firing versus spontaneous rate (A) and first-spike latency (B). Legend in A also applies to B.

Neurons with low spontaneous rates did not necessarily have a low probability of firing in response to a click (Fig. 6A). While neurons showing low probability of firing tended to have low spontaneous rates (<10 Hz), neurons with a high probability of firing could also have very low spontaneous rates. In particular, medial MGB neurons displayed high response probabilities even at the lowest spontaneous rates.

Fig. 6 also shows the relationship between probability of firing to a click and the neurons’ first-spike latency to that stimulus (Fig. 6B). In the case of the medial MGB, those neurons which showed the highest probability of response tended to have shorter response latencies (between 5 and 14 ms); however not all medial MGB neurons which had shorter response latencies showed high probability of firing. This pattern was not so apparent for the other subdivisions.

#### 3.3.4 Temporal response properties

**Fig. 7:**
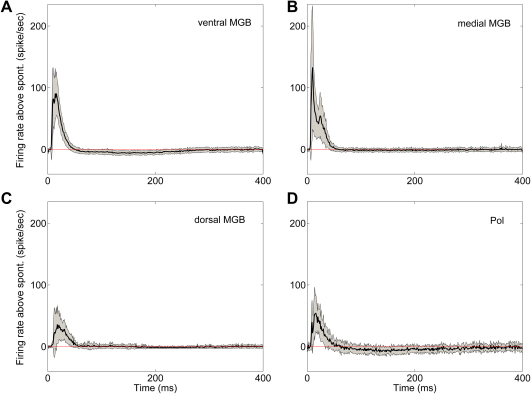
Mean PSTH of response to click stimulus, normalised for each neuron by subtracting spontaneous rate, for ventral MGB (A), medial MGB (B), dorsal MGB (C) and Pol (D). Black line indicates mean response; grey area indicates range of standard error of mean.

Observation of the population PSTHs to click normalised by the spontaneous rate (see Fig. 7) revealed the firing rate immediately returned to baseline following the response in the medial (Fig. 7B) and dorsal MGB (Fig. 7C), whereas in the ventral MGB (Fig. 7A) and Pol (Fig. 7D) the firing rate dipped slightly below spontaneous rate for the period of 75–275 ms after stimulus onset. The presence of higher spontaneous rates in the ventral MGB and Pol may have made the dip below spontaneous rate more apparent than for the medial and dorsal MGB. However, while the dip below spontaneous rate was apparent at the single cell level in the ventral MGB and Pol, it was not observed in single cells in medial and dorsal MGB, even those which showed higher spontaneous rates.

**Fig. 8:**
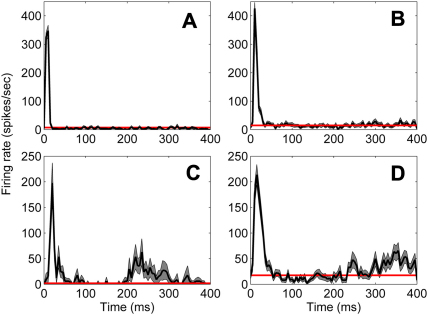
Temporal response characteristics for individual neurons, illustrated by plotting the mean PSTH (black line) and standard error of the mean (grey shading). Spontaneous rate for each cell is indicated by the red line. A, PSTH from a single cell recorded from the medial MGB illustrating an “onset-only” response to the click stimulus. B, PSTH as in A recorded from the ventral MGB. C, PSTH from a single cell from the ventral MGB showing a significant increase in activity at 200 ms following the initial response which returns to baseline by 400 ms. D, PSTH from a single cell also from the ventral MGB showing a significant increase in activity from 300 ms after stimulus onset. The later activity of this cell remains significantly above baseline at the end of the recording.

Observation of the PSTHs in response to a click on a cell-by-cell basis showed that while the majority of neurons simply showed an onset response to the click stimulus (examples shown in Fig. 8A and B), some appeared to show a subsequent later increase in activity, over-and-above the spontaneous rate, regardless of whether or not the activity had previously dropped below spontaneous rate (Fig. 8C and D). This increased late firing did not appear to be oscillatory; there were no instances of multiple regular periods of excitation alternating with periods of low-discharge probability within the recorded interval. The time course of the increase in activity, which was significantly greater than the baseline spontaneous rate (determined using a randomisation test, see Methods), varied between neurons but typically occurred ∼250 ms after stimulus onset and could continue for up to at least 400 ms after the stimulus onset when recording ended. This variable time course is illustrated in the two neurons in Fig. 8C and D; the neuron in Fig. 8C shows an increase in firing during the period 200–300 ms after stimulus onset, which returns to the baseline spontaneous rate by 400 ms, whereas the example in Fig. 8D shows a consistent later increase in firing from 300 ms which does not return to the baseline during the recording period. The examples in Fig. 8C and D also illustrate the variability of firing during this later period compared to the precision of the response to the click stimulus (note the difference in grey shading over the initial click response and the later firing). Neurons showing this later response were exclusively located either in the ventral MGB (88%) or the Pol (12%), where they accounted for 28% of the ventral MGB population and 20% of the Pol population. Neurons in the medial and dorsal MGB did not have firing rates in this later period that were significantly greater than the spontaneous rate.

### 3.4 Response to tonal stimuli

#### 3.4.1 Characteristic frequency and threshold distribution

**Fig. 9:**
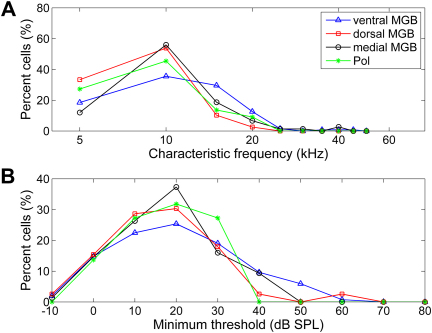
Characteristic frequency and minimum threshold distributions for different auditory thalamic subdivisions. Line plots show the percentage distribution of CF (A) and minimum threshold (B) values for the ventral, dorsal and medial MGB and the Pol.

Minimum threshold (the lowest sound level at which the neuron responds to tonal stimuli) and characteristic frequency (CF, the frequency at which the neuron responds at the minimum threshold) were measured for each neuron with a clear response to tonal stimuli. The distributions of CFs and minimum thresholds recorded across the different subdivisions are shown in Fig. 9A and B respectively. The distributions of minimum thresholds were not significantly different across the subdivisions (Kruskal-Wallis test, *p* > 0.1). The distributions of CFs recorded from the Pol, ventral and medial MGB were not significantly different (Kruskal-Wallis test, *p* > 0.1), however, the range of CFs in the dorsal MGB was significantly narrower than that observed in the medial and ventral MGB and the Pol (Kruskal-Wallis test, *p* < 0.01). This is unlikely to be due to the presence of a greater number of untuned neurons in the dorsal MGB, as a similar proportion of untuned neurons were also recorded from the Pol (see Fig. 11). The different distribution of CFs in the dorsal MGB compared to the other subdivisions could not account for the differences in other physiological response properties. Significant differences in response latency and probability were observed between the different subdivisions regardless of whether these response measures were estimated using data from the full CF range or a CF range restricted to be the same for each subdivision.

#### 3.4.2 Tuning and frequency-intensity response area shape

**Fig. 10:**
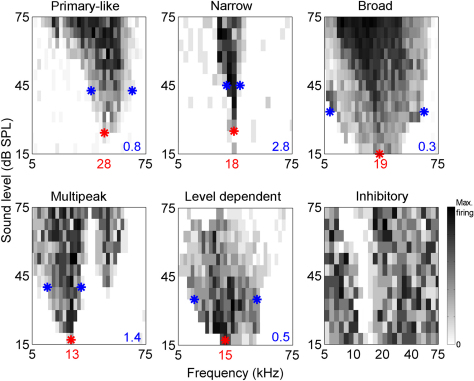
Example frequency-intensity response area shapes recorded from single neurons in the mouse auditory thalamus. All response areas shown were compiled using tone pips varying in frequency from 5 to 75 kHz and in sound level from 15 to 75 dB SPL. Red asterisk and frequency shown in red on the abscissa indicates the characteristic frequency for each neuron. Blue asterisks indicate the low and high frequencies used to calculate the Q20; Q20 values for each neuron are shown in blue in the lower right corner of each plot. Primary-like, level-dependent and inhibitory examples taken from the ventral MGB, narrow neuron from Pol, broad neuron from dorsal MGB, multipeak neuron from medial MGB. Maximum firing for inhibitory example = 20 spikes/sec, all other examples = 100 spikes/sec.

**Fig. 11:**
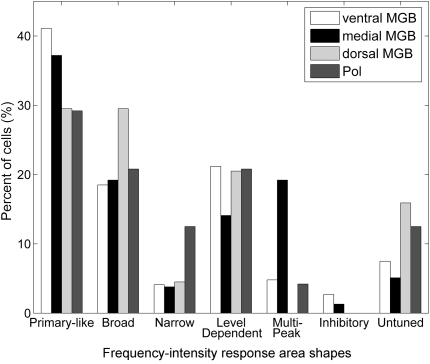
Distribution of different frequency-intensity response area shapes in the different auditory thalamic subdivisions.

Six different frequency-intensity response area shapes were routinely recorded from the mouse MGB: primary-like, narrow, multipeaked, level-dependent, broad and inhibitory (examples of each are shown in Fig. 10). Neurons which responded to the broadband stimuli but which showed no consistent increase in firing rate to any frequency/level combination were classed as untuned; no characteristic frequency was obtainable for these neurons. The most common response type observed across the whole MGB was the primary-like, single-peaked response, which accounted for 46% for the units sampled. The distribution of the frequency-intensity response area types varied between the subdivisions as shown in Fig. 11. All subdivisions showed a considerable degree of overlap in the types of response area observed, although slight differences between the subdivisions were noticeable, e.g. the medial MGB contained a greater proportion of multipeaked responses compared to other auditory thalamic subdivisions, the Pol showed a greater proportion of narrow response areas compared to the other areas and the dorsal MGB contained a greater number of broadly-tuned neurons. Both the ventral and medial MGB were dominated by the primary-like response area which made up 41% and 37% of the response area shapes recorded from these subdivisions. Conversely, the response area shapes in the dorsal MGB and Pol were more evenly distributed, with primary-like, broad and level-dependent shapes each accounting for approximately one third of the distribution.

The bandwidth of the tuning curve normalised by CF was measured at three different levels (10, 20 and 40 dB above threshold [Q10, Q20, and Q40]). The distributions of Q10, Q20 and Q40 values were significantly different between the dorsal and ventral MGB (Kruskal-Wallis test, *p* < 0.01), and between the dorsal and medial MGB for the Q20 and Q40 values (Kruskal-Wallis test, *p* < 0.01). Interestingly, there was no significant difference in the distribution of Q values between the medial MGB, ventral MGB and Pol (Kruskal-Wallis test, *p* > 0.1). Calculations of bandwidth using the square root transformation √f2−√f1, where f2 and f1 indicate the high and low limits of the tuning curve bandwidth 10, 20 or 40 dB above the minimum threshold, showed the same trends as the Q values.

## 4 Discussion

### 4.1 Summary of findings

As in previous auditory thalamic studies carried out in a range of species (Anderson et al., 2007; Calford, 1983; Redies and Brandner, 1991), response latencies recorded from the dorsal MGB of the mouse were considerably slower than those recorded from any of the other auditory thalamic subdivisions. (Indeed, with the peak of the first-spike latency distribution occurring some 18 ms later in the dorsal MGB compared to the ventral MGB, the dorsal MGB response latencies were much slower in the mouse than observed in other species for the same stimulus (Anderson et al., 2006).) Conversely, medial MGB neurons had on average significantly shorter response latencies to a broadband click than any of the other auditory thalamic subdivisions, including the ventral MGB. Short-latency responses in medial MGB have previously been reported in the cat (Rouiller et al., 1989), guinea pig (Anderson et al., 2006) and rat (Bordi and LeDoux, 1994a). Response latencies in the ventral MGB and Pol were not significantly different from each other.

Neurons in the medial MGB were significantly more likely to respond to the click stimulus than neurons in either the dorsal MGB or the Pol. There was no significant difference in response probability between medial and ventral MGB; however a greater proportion of medial than ventral MGB neurons were capable of responding to 100% of the stimulus repetitions. This was despite the fact that medial MGB neurons had a significantly lower spontaneous firing rate than ventral MGB neurons. Thus, the neurons’ probability of firing to the click stimulus could not be predicted from the spontaneous rate alone. Indeed, despite significant differences in the probability of firing to the click, neurons in the medial and dorsal MGB had very low spontaneous rates which were not significantly different between the two subdivisions. Conversely, neurons in the ventral MGB and Pol had higher spontaneous rates which were not significantly different from each other, but the two subdivisions showed significant differences in their probability of firing.

The subdivision differences in response latency and probability of firing were evident not only at the population level, but also in single cells. As has been shown in other species, clear changes in response properties were frequently encountered as the electrode crossed between subdivision boundaries (Anderson et al., 2007 [guinea pig]; Calford, 1983 [cat]). Intracellular in-vitro studies have shown that in comparison to ventral and dorsal MGB neurons, medial MGB neurons are more excitable, have larger action potentials and do not exhibit the calcium bursting common to the ventral and dorsal MGB (Smith et al., 2006). Increased excitability might underlie the ability of the medial MGB to respond to the broadband click with shorter latencies than the other subdivisions, but it is unclear how it could co-exist with very low spontaneous firing rates.

Neurons in ventral MGB and Pol not only had higher spontaneous rates than neurons in medial and dorsal MGB, but also increased probability of firing in a late period ∼250 ms after the click. In principle, this late “rebound” of activity might also exist in medial and dorsal MGB neurons, but be more difficult to detect in extracellular recordings, for example if medial and dorsal MGB neurons had higher response thresholds. However, neuronal tone response thresholds in medial MGB were similar to those in ventral MGB, and thresholds in dorsal MGB were similar to those in Pol (Table 1). Therefore, it seems possible that the increased probability of late firing is a characteristic of neurons in the lemniscal pathway. “Reverberatory” activity has previously been described in lemniscal MGB of cat, although the late increase in firing observed here in the mouse occurred after a longer period of suppression, and did not show the regular oscillating patterns of excitation and inhibition observed in the cat (Aitkin et al., 1966; Ryugo and Weinberger, 1976).

Differences in responses of the auditory thalamic subdivisions to tonal stimuli were more subtle than the differences in click responses. Frequency-intensity response areas were divided into shape categories previously suggested for the MGB: primary-like (V-shaped), narrow, broad, and multipeaked (Anderson et al., 2007; Morel et al., 1987; Rodrigues-Dageff et al., 1989; Rouiller et al., 1989). We also identified level-dependent and inhibitory response shapes which were probably categorised as atypical in previous studies.

The medial MGB has previously been reported to contain a higher proportion of broadly-tuned neurons than the other MGB subdivisions (Aitkin, 1973; Edeline et al., 1999; Rouiller et al., 1989). Interestingly, we found this not to be the case in the mouse; there was no significant difference between the medial and ventral MGB in either Q values or √f2−√f1 values for frequency tuning at a range of different sound levels. The observation of narrower response areas in the medial MGB in this present study compared to the studies of Rouiller et al. (1989) and Edeline et al. (1999) could arise from differences in anaesthesia. These two previous studies used either lightly-anaesthetised (nitrous-oxide, (Rouiller et al., 1989)) or awake (Edeline et al., 1999) preparations, whereas we used a moderate anaesthetic regime of ketamine and medetomidine. At the level of the auditory thalamus, ketamine has been shown to decrease bandwidth compared to the awake or lightly-anaesthetised preparations (Zurita et al., 1994). However, broad tuning in the cat medial MGB was also observed under pentobarbital anaesthesia (Aitkin, 1973) which at the level of the auditory thalamus has been shown to have a similar effect on frequency selectivity as ketamine anaesthesia (Zurita et al., 1994). To confirm that the similarities observed here between the ventral and medial MGB were not caused by differences in methods for measuring neuronal bandwidth, we also measured bandwidth as per Aitkin (1973) but still saw no significant difference in bandwidth between the medial and ventral MGB (Kruskal-Wallis test, *p* > 0.1, data not shown). Thus, the divergent findings on bandwidth observed between the current and previous studies likely arise from differences in species rather than differences in anaesthetic conditions or analysis methods.

The only subdivision to show a significant difference in bandwidth from the others was the dorsal MGB, which had a greater proportion of broadly-tuned response areas compared to the other auditory thalamic areas. There was no difference in minimum threshold between any of the auditory thalamic subdivisions. Therefore, given the degree in overlap between auditory thalamic subdivisions in distributions of characteristic frequency, minimum threshold, response area shape and bandwidth, precise on-line assignment of neurons to a particular subdivision was not possible on the basis of basic tonal response properties alone.

We did not attempt to unravel the tonotopic organisation of the MGB subdivisions from single electrode penetrations; however another study in the mouse MGB using multielectrodes has shown that the ventral MGB has a complex tonotopic organisation (Polley et al., 2010), as has been reported in a number of other species (Anderson et al., 2007; Bordi and LeDoux, 1994a; Morel et al., 1987). A frequency organisation of the medial MGB has been proposed in the cat (Rouiller et al., 1989) but remains to be fully elucidated in smaller species, although there have been suggestions that a weak tonotopy exists in the medial MGB of the rat (Bordi and LeDoux, 1994a).

Increased metabolic activity in the ventral and medial MGB as indicated by the distribution of cytochrome oxidase (CYO) across the MGB was consistent with the physiological observations of high response probabilities and short response latencies in both the ventral and medial subdivisions. The ventral and medial MGB both showed high levels of CYO expression which clearly delineated them from the dorsal MGB which had very low levels of CYO; intermediate levels of CYO were observed in Pol. The levels of CYO expression in the medial MGB were higher than observed previously (Gonzalez-Lima and Cada, 1994); this difference may be due to slight differences in the staining technique or genetic background of the mouse strain. The higher activity in the medial MGB served to enhance the medial/dorsal MGB border, while a band of paler staining distinguished the medial MGB from the ventral MGB. These staining patterns corresponded well with those seen in other species (Anderson et al., 2007; Cant and Benson, 2007; Hackett et al., 1998).

### 4.2 Auditory thalamocortical pathways

The terms lemniscal and non-lemniscal were originally proposed to describe areas of the auditory thalamus that received an anatomical projection from either the central nucleus of the inferior colliculus (lemniscal) or the external collicular nuclei (non-lemniscal). The lemniscal thalamocortical projection arises from the ventral MGB, which receives the majority of its afferent input from the central nucleus of the inferior colliculus (IC) (Andersen et al., 1980b; Calford and Aitkin, 1983; Winer, 1992 [review]), whereas the non-lemniscal thalamocortical projection originates from a group of nuclei surrounding the ventral MGB, including the dorsal and medial MGB and the Pol, which were considered to receive the majority of their afferent innervatation from the external and lateral nuclei of the IC (Andersen et al., 1980b; Calford and Aitkin, 1983; Winer, 1992 [review]). Other anatomical studies have suggested that non-lemniscal areas are characterised as those which have a strong efferent projection to the amygdala (Doron and LeDoux, 2000; Linke, 1999) or those which lack a direct connection to the sensory periphery, instead receiving projections from the shell and external nuclei of the IC, sagulum and spinothalamic tract (Winer, 1992 [review]). The lemniscal and non-lemniscal areas have also been reported to show histological differences, including a differing affinity for the calcium-binding proteins parvalbumin and calbindin (Cruikshank et al., 2001; Lu et al., 2009), and differences in their cytochrome oxidase distribution (Gonzalez-Lima and Cada, 1994).

The presence of different anatomical inputs led to the proposal that the lemniscal and non-lemniscal pathways have largely different functional capabilities. The lemniscal projection is tonotopically organised, and capable of conveying high-fidelity timing and tuning information, whereas the non-lemniscal pathway is thought to play a more important role in the representation of sound context, such as the behavioural significance of a sound or its probability of occurrence based on recent stimulus history. This proposal is supported by previous studies of single cell recordings from the ventral and dorsal MGB (Anderson et al., 2007 [guinea pig]; Calford and Aitkin, 1983 [cat]), as well as the current study.

However, neurons in the medial MGB and Pol do not fit this dichotomy so neatly. The medial MGB seems to have characteristics of both the ventral and dorsal MGB. Histologically, the lemniscal and non-lemniscal areas of the auditory forebrain can be delineated using the calcium-binding proteins parvalbumin (lemniscal) and calbindin (non-lemniscal) (Cruikshank et al., 2001; Lu et al., 2009). However, the medial MGB not only contains a significant proportion of cells which show high immunoreactivity for calbindin (also present in high levels in dorsal MGB), as would be expected if the medial MGB were part of the non-lemniscal pathway, but also cells which contain parvalbumin (present in high levels in ventral MGB) (Cruikshank et al., 2001; Lu et al., 2009) and calretinin, a calcium binding protein not commonly observed in other areas of the MGB (Lu et al., 2009).

As shown here for the mouse and previously in a number of other species, in addition to its anatomical heterogeneity the medial MGB contains cells which show a variety of physiological response properties encompassing those seen in both the ventral and dorsal MGB (Aitkin, 1973; Anderson et al., 2007; Anderson et al., 2006; Bordi and LeDoux, 1994a; Calford and Aitkin, 1983; Edeline et al., 1999). Moreover, responses have been recorded from the medial MGB that exceed the observed capabilities of neurons even in the lemniscal ventral MGB in terms of fast response latency (Anderson et al., 2006; Rouiller et al., 1989) and enhanced temporal following (Anderson et al., 2005; Rouiller et al., 1979; Rouiller et al., 1981; Wallace et al., 2007). The medial MGB has also been reported to be tonotopically organised at least in part (Bordi and LeDoux, 1994a; Rouiller et al., 1989), making it more similar to the ventral MGB than the non-lemniscal dorsal MGB. Conversely, medial MGB neurons have an enhanced ability to detect change in auditory signals (Anderson et al., 2009b; Baeuerle et al., 2009; Kraus et al., 1994) compared to the ventral MGB, a response feature suggested to be more characteristic of the non-lemniscal than lemniscal areas.

Likewise, the current study shows the Pol to have response latencies, spontaneous rates and temporal response patterns which are not significantly different from those of the ventral MGB. Previous studies on response properties of the Pol have highlighted its high proportion of primary-like frequency-intensity response areas (Phillips and Irvine, 1979); our recordings showed a wider distribution of frequency-response area shapes in the Pol, including a high proportion of primary-like shapes and greater proportion of narrow response shapes than the other auditory thalamic subdivisions. Overall the bandwidths of Pol neurons were not significantly different from those recorded in ventral MGB. The presence of a tonotopic organisation in the Pol of the cat (Imig and Morel, 1985) also supports the fact that the Pol is more similar to the ventral MGB than to the non-tonotopically organised, more broadly-tuned, non-lemniscal dorsal MGB.

The heterogeneity of both the connectivity and the physiological response patterns recorded from the medial MGB (Winer, 1992), alongside the similarity of the Pol and ventral MGB in terms of their response properties and connectivity (de-Ribaupierre, 1997; Imig and Morel, 1985), have prompted suggestions for a revision of the traditional lemniscal/non-lemniscal subdivision of the auditory thalamocortical pathways into at least three different afferent projections. Each of the three projections, the “*tonotopic*”, “*non-tonotopic or diffuse*”, and the “*polysensory*”, encompasses one of the major MGB subdivisions. The tonotopic pathway originates in the central nucleus of the IC, incorporates the ventral MGB and Pol, and continues to the primary auditory cortices (Calford and Aitkin, 1983; de-Ribaupierre, 1997) where the afferent neurons terminate primarily in layers III/IV (Kimura et al., 2003; Winer et al., 2005). The diffuse or non-tonotopic pathway incorporates the dorsal MGB, which receives afferent projections from the dorsal and lateral cortices of the IC and projects to the secondary auditory cortices with afferent neurons terminating across layers I, II, III/IV and VI (Kimura et al., 2003; Winer et al., 2005). This pathway may also include a cortico-collicular loop enabling the response to auditory stimuli to be modulated by higher-order (cortical) processing (Calford and Aitkin, 1983; Lee and Sherman, 2010). The varied physiological responses recorded from the medial MGB likely result from the variety of afferent inputs it receives, not only from all parts of the IC (Andersen et al., 1980b; Calford and Aitkin, 1983; Kudo and Niimi, 1980) but also the ventral nucleus of the lateral lemniscus (Whitley and Henkel, 1984) and dorsal cochlear nucleus (Anderson et al., 2006; Anderson et al., 2009c; Malmierca et al., 2002), as well as many non-auditory areas (Winer, 1992 [review]). These observations have led to proposals for the medial MGB to be considered part of a separate third thalamocortical pathway, the polysensory pathway, which projects to all parts of the auditory cortex across a variety of layers, with strong projections to layers I and VI as well as layers III and IV (Calford and Aitkin, 1983; de-Ribaupierre, 1997; Kimura et al., 2003; Winer et al., 2005). While these pathways connect distinct regions of the auditory thalamus with the auditory cortex, it is important to note that at each level interconnections exist between the three systems (de-Ribaupierre, 1997).

Here, we have shown that the ventral and dorsal subdivisions of the mouse MGB have clearly different physiological response properties, as predicted by the traditional classification of these areas into lemniscal and non-lemniscal regions. However, despite frequently being classified as a non-primary or non-lemniscal area, the medial MGB contains neurons with physiological response properties that exceed not only the properties typical of a non-lemniscal area, but also those of the lemniscal ventral MGB. Moreover, response properties of Pol are more similar to those of ventral MGB than dorsal MGB. Thus, we suggest that a tripartite subdivision of the ascending thalamocortical pathways represents a more appropriate description of the mouse auditory thalamus than the more traditional dichotomy between high-fidelity lemniscal and context-dependent non-lemniscal pathways.

## Figures and Tables

**Table 1:** Summary of click and tone response characteristics of neurons recorded from the MGB subdivisions and Pol. Where appropriate mean values are shown ± standard error.

	Ventral MGB	Dorsal MGB	Medial MGB	Pol
Median first-spike latency (ms)	14.9	27.5	11.7	17.1
First-spike latency inter-quartile range (ms)	9.2	14.7	6.0	12.7
Min. first-spike latency (ms)	6	10.5	5.5	7.8
Max. first-spike latency (ms)	60.5	71.8	48.5	40.8
Mean spontaneous rate (spikes/sec)	8.8 ± 0.8	2.2 ± 0.3	2.6 ± 0.6	9.59 ± 1.8
Mean probability of firing	0.71 ± 0.04	0.55 ± 0.07	0.74 ± 0.05	0.62 ± 0.09
Proportion showing increased late firing (%)	28	0	0	20
CF range (kHz)	5.1–45.5	5.6–20.0	7.2–51.9	5.2–36.5
Mean threshold (dB SPL)	23 ± 1.2	30 ± 2.2	20.5 ± 1.4	30 ± 2.1
FRA response shape (%)				
Primary-like	41.1	29.5	37.2	29.2
Narrow	4.1	4.5	3.8	12.5
Broad	18.5	29.5	19.2	20.8
Multipeaked	4.8	0	19.2	4.2
Level-dependent	21.2	20.5	14.1	20.8
Inhibitory	2.7	0	1.3	0
Untuned	7.5	15.9	5.1	12.5
Mean bandwidth (dB)				
Q10	2.1 ± 0.1	2.4 ± 0.2	2.4 ± 0.2	2.3 ± 0.3
Q20	1.4 ± 0.1	1.7 ± 0.1	1.4 ± 0.1	1.6 ± 0.2
Q40	1.3 ± 0.1	1.5 ± 0.3	1.3 ± 0.2	1.2 ± 0.2
